# Survivorship therapy needs after radiotherapy for head and neck cancer: surveying opportunities for growth (STRONG)

**DOI:** 10.1007/s00520-025-09429-2

**Published:** 2025-04-29

**Authors:** Alexis Larson, Yangruijue Ma, Zequn Sun, Janine Kingsbury, Katelyn O. Stepan, Adil Akthar, Jochen Lorch, Bharat B. Mittal, Poonam Yadav, Michelle L. Mierzwa, Leila J. Mady, Laila A. Gharzai

**Affiliations:** 1 Department of Radiation Oncology, Northwestern University, 251 East Huron Street, Ste LC- 178, Chicago, IL 60611 USA; 2 Department of Biostatistics, Northwestern University, Chicago, IL USA; 3 Department of Otolaryngology, Northwestern University, Chicago, IL USA; 4 Division of Medical Oncology, Department of Medicine, Northwestern University, Chicago, IL USA; 5 Department of Radiation Oncology, University of Michigan, Ann Arbor, MI USA; 6 Department of Otolaryngology, Johns Hopkins University, Baltimore, MD USA; 7 Department of Medical Social Sciences, Northwestern University, Chicago, IL USA

**Keywords:** Head and neck cancer, Radiotherapy, Survivorship

## Abstract

**Introduction:**

Patients with head and neck squamous cell carcinoma (HNSCC) are at risk for long-term side effects and require thorough survivorship care following completion of radiation therapy (RT). We aimed to identify survivorship interest and needs following RT.

**Methods:**

Patients with HNSCC who completed RT from Jan 2013 to April 2023 completed questions based on the five-domain Cancer Survivorship Framework (physical effects, psychosocial effects, cancer screening, chronic conditions, health promotion) and the Consolidated Framework for Implementation Research (interest, knowledge, barriers).

**Results:**

Of 1,123 patients, 317 participated (response rate 28%). Patients were 64% (*n* = 203) male with a mean age of 64 years (SD 12), with 66% (*n* = 209) finishing treatment > 2-years prior. 36% (*n* = 115) were interested in, 34% (*n* = 108) prioritized, and 28% (*n* = 88) had information related to survivorship. For survivorship domains, within physical effects, dry mouth (40%, *n* = 126) and trouble swallowing (24%, *n* = 75) were most bothersome. For psychosocial, 15% (*n* = 46) reported current depression (mean PHQ2 score ≥ 3) and 71% (*n* = 226) felt mental health needs were not addressed during treatment. Forty-two percent felt that diet, exercise, and smoking were not addressed (*n* = 134). Seventy-six percent (*n* = 240) understood the importance of cancer screening. Most patients wished to address survivorship topics in one long visit (45%, *n* = 142) and preferred in-person visits (60%, *n* = 190). Potential barriers included insurance coverage and scheduling concerns.

**Conclusion:**

In post-RT HNSCC survivors, survivorship interest and knowledge were limited. There is a need for education on survivorship.

**Implications:**

These findings provide a foundation for developing robust survivorship care programs that meet the diverse needs of this patient population.

**Supplementary Information:**

The online version contains supplementary material available at 10.1007/s00520-025-09429-2.

## Introduction

The number of long-term survivors of head and neck squamous cell carcinomas (HNSCC) has been increasing due to more efficacious treatments and changes in disease epidemiology. The influence of human papillomavirus (HPV) on the development of oropharyngeal cancers has now led HPV-related HNSCC to be the most commonly diagnosed HNSCC [1–3], with a higher tumor response and survival rate as compared to HPV-negative HNSCC, which is more commonly associated with smoking or tobacco use [4–6]. Five-year survival rates now range from approximately 60–80% for non-metastatic patients [7], and the number of patients living long-term after treatment continues to rise.

Patients with HNSCC are often exposed to multimodal treatment, with many patients undergoing combinations of surgery, radiation therapy (RT), and systemic therapy [8]. RT is an important component of management for HNSCC, both in the definitive and postoperative settings [9]. As a result, patients are at risk for many short and long-term side effects from RT, ranging from mucositis during treatment or long-term xerostomia and dysphagia after treatment [10–15]. These long-term effects can negatively impact the patient’s ability to function, overall quality of life, and mental and emotional well-being [16–19]. Thus, HNSCC survivors have a wide range of needs post-treatment.

A number of survivorship guidelines for HNSCC exist [20–23], but none comprehensively address wide ranging HNSCC-related needs, and it remains challenging to implement a survivorship clinic that addresses the vast needs of this growing population [24–27]. There is an unmet need to understand survivor needs in the context of a validated survivorship framework assessing both specific needs and overall insights into how to optimize a survivorship program [28]. In this cross-sectional survey study, we sought to utilize a comprehensive survivorship framework to gain insight on needs and preferences of survivorship care post-RT for patients with HNSCC.

## Methods

### Study participants and data collection

Patients with HNSCC who had undergone RT from January 2013 to April 2023 were eligible to participate in this anonymous survey. Eligible patients were identified through Northwestern University’s institutional data repository, and those with a valid email address collected from the electronic medical record were included. No data was collected from the electronic medical record other than email addresses. The electronic survey was sent via email on June 20, 2023, with additional reminders sent on June 30 and July 10, 2023. The survey was closed on July 27, 2023. This anonymous survey was deemed exempt by the Northwestern University Institutional Review Board (STU00218427).

### Measures

Study participants completed a 20-min, 47-item online survey assessing cancer survivorship domains and barriers/facilitators to participating in a survivorship clinic. Study data were collected and managed using the web-based Research Electronic Data Capture (REDCap) tool hosted at Northwestern University Feinberg School of Medicine [29]. Cancer survivorship domains were based on Nekhlyudov et al.’s Cancer Survivorship Framework, which encompasses five domains (Supplementary Fig. 1): (1) physical effects, (2) psychosocial effects, (3) cancer screening, (4) management of chronic conditions, and (5) health promotion [24]. The measure was developed by a multi-disciplinary study team using standardized approaches to questionnaire design [30], and after development the survey was reviewed by five additional people (three HNSCC survivors and two radiation oncology nurses).

A total of 22 questions covered the five survivorship domains. We adapted existing measures relevant to the domains of interest and developed new questions when needed. Ten questions on ongoing physical effects were adapted from the Mayo Clinic Cancer Center Survey of Cancer Survivors [31]. Three questions on psychosocial effects included the Patient Health Questionnaire- 2 (PHQ- 2) [32], with a score of ≥ 3 defining depression, and an additional item developed by the study team asking whether mental health needs were addressed throughout their cancer journey. Three questions developed by the study team on health promotion included asking participants if their cancer providers discussed the importance of exercise, healthy diet, and smoking cessation. One item on chronic conditions asked about confidence in managing chronic conditions with their primary care physician, and one item on cancer screening asked about the importance of screening; these were both developed by the study team.

To address contextual factors impacting survivorship, we utilized the Consolidated Framework for Implementation Research (CFIR) domains [33] and developed three questions from domains deemed relevant by study team members with expertise in implementation science. These included the individual domain (assessing interest in survivorship), inner setting process domain (access to information in survivorship), implementation process domain (considering survivorship as a priority). Finally, specific questions were developed to assess preferred structure of a survivorship clinic, patient preferences on visit frequency, number of topics they would like to address, and modality of interaction (i.e., inclusion of telehealth options). The remainder of the survey consisted of questions on demographics (age, gender, race, zip code) and cancer treatment received (currently undergoing treatment, received surgery, received chemotherapy, received RT).

### Statistical analysis

The primary aim of this analysis was to descriptively assess gaps in survivorship care and aid in the design of a comprehensive survivorship clinic for patients with HNSCC. For items on physical effects, responses on a scale of 0 (“No concern”) to 5 (“Great deal of concern”) were dichotomized, with scores of 4 or 5 classified as “yes” to having a bothersome level of concern. For CFIR domains, a Likert scale from strongly disagree to strongly agree was dichotomized with agree and strongly agree classified as “yes”. For items on health promotion (exercise, healthy diet, smoking cessation), responses were analyzed separately and combined into a composite variable. Patients who responded “yes” to all three items were classified as having received comprehensive counseling on health promotion. Descriptive statistics were used to summarize the responses of the survey for the overall study population and for each subgroup. We performed a multivariate analysis (MVA) assessing predictors of the CFIR questions (interest, access, priority) and then further performed two prespecified subgroup analyses by gender and duration from treatment (≤ 2 years versus > 2 years, based on current guidelines to decrease frequency of oncologic surveillance after 2 years[9]). Continuous variables were summarized using median and interquartile range (IQR) and were compared between groups using the Wilcoxon rank sum test. For categorical variables, frequencies and percentages were reported, and proportions were compared using Fisher’s exact test. All statistical tests were two-sided, and a *p*-value of less than 0.05 was considered statistically significant. All analyses were conducted in R Version 4.3.1. Data is available upon reasonable request from the corresponding author.

## Results

Of the 1,123 potential participants who underwent RT for HNSCC and had valid email addresses, 343 started the survey. Of these, 26 did not answer any questions, leaving 317 patients eligible for analysis, resulting in a 28% response rate (Supplementary Fig. 2). Among these participants, 64% were male (*n* = 203) with a mean age of 64 years (SD 12). Two thirds (*n* = 209) had completed treatment more than 2 years prior to the survey. In terms of ethnicity, 81% of participants were non-Hispanic White (*n* = 256). At the time of the survey, 79% reported that their cancer had not returned since completing RT (*n* = 251). Regarding additional treatment modalities in these post-RT patients, 69% of participants had undergone surgery (*n* = 219), and 49% received chemotherapy (*n* = 155) as part of their cancer treatment (see Table 1).

**Table 1:** Demographics

	**n**	**%**
Age (mean, SD)	64	(12)
Gender		
Male	203	64.0%
Female	81	26.0%
Missing	33	10.0%
Race		
White	256	81.0%
Black	7	2.2%
Asian Americans, Native Hawaiians, and Pacific Islanders	12	3.9%
Other, Prefer not to Answer, or Missing	42	13.3%
Treatment Received		
Radiotherapy	317	100.0%
Chemotherapy	155	49.0%
Surgery	219	69.0%
Length Since Radiation Treatment		
≤ 2 years	86	27.1%
> 2 years	209	66%
Missing	22	6.90%
Cancer Recurrence	38	12%
Currently undergoing treatment	26	8.20%

**Fig. 1:**
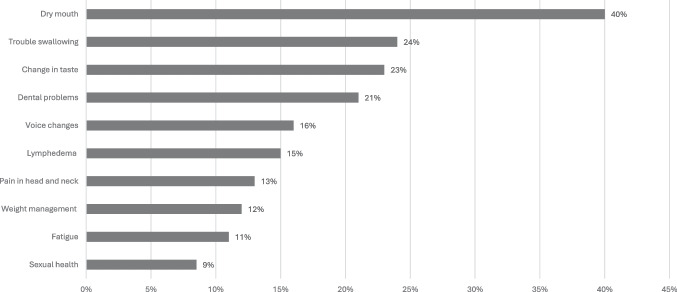
Ongoing physical effects reported by HNSCC survivors (*n* = 317)

For the five survivorship domains, the most reported physical symptoms (with > 20% participant endorsement) were dry mouth in 40% (*n* = 126), trouble swallowing in 24% (*n* = 75), taste changes in 23% (*n* = 72), and dental problems in 21% (*n* = 66) (see Fig. 1). In the psychosocial domain, 15% (*n* = 46) of participants scored PHQ2 ≥ 3, indicating depression. Seventy-one percent (*n* = 226) felt their mental health was not addressed during their cancer journey.

For cancer screening, 12% (*n* = 38) stated that their cancer providers did not discuss the importance of screenings. In terms of health promotion, a combined 42% (*n* = 134) reported not discussing the benefits of smoking cessation (*n* = 15, 4.7%), diet (*n* = 68, 21%), and exercise (*n* = 87, 27%) with their cancer providers. Additionally, 12% (*n* = 38) of participants were unsure or not confident in managing their chronic conditions with their primary care physician.

When asked about knowledge related to survivorship, 36% (*n* = 113) of participants expressed interest in survivorship care, and 34% (*n* = 108) stated it was a priority, but only 28% (*n* = 88) reported having access to information about survivorship care. On MVA, female patients were more likely to express interest in survivorship care (OR 2.67, 95%CI 1.48–4.86, *p* < 0.001). Similarly, female patients were more likely to state survivorship care is a priority (OR 2.79, 95%CI 1.54–5.11, *p* < 0.001); patients who completed treatment > 2 years prior were less likely to state survivorship care is a priority (OR 0.52, 95%CI 0.29–0.93, *p* = 0.027). Patients who had undergone multimodality treatment or were currently undergoing treatment were more likely to have access to survivorship care (received surgery OR 2.50 (95%CI 1.24–5.29, *p* = 0.013), received chemotherapy (OR 2.09 (95%CI 1.15–3.85, *p* = 0.016), currently undergoing treatment OR 3.01 (95%CI 1.08–8.83, *p* = 0.038). Patients who completed treatment > 2 years prior were less likely to have access to survivorship care (OR 0.34, 95%CI 0.19–0.62, *p* < 0.001, Supplementary Table 1).

**Fig. 2:**
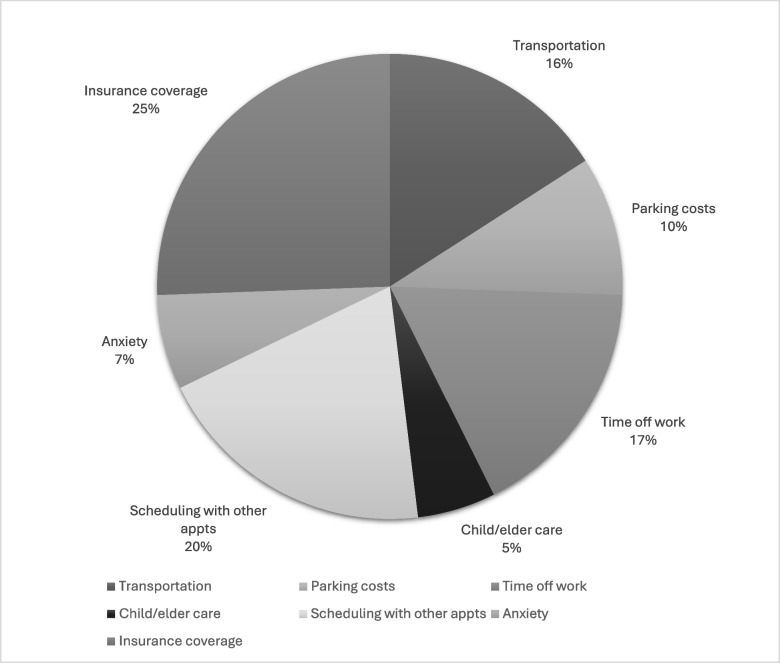
Barriers to attending survivorship visits

Regarding survivorship clinic preferences, 45% (*n* = 142) preferred one longer visit to address all topics rather than multiple shorter visits. Sixty percent (*n* = 190) favored in-person follow-up visits, while 39% (*n* = 123) preferred telehealth visits. Thirty-one percent (*n* = 99) of participants were interested in attending survivorship visits once a year followed by 23% (*n* = 72) every 6 months. Twenty-three percent (*n* = 73) were not interested in attending survivorship visits (see Table 2), and when given the option to free-type in “other,” 4 stated they already see their other physicians throughout the year and they do not see the need. We then asked about barriers to attending survivorship visits. Participants were given the option to select multiple barriers or none of the above. Of those that responded, insurance coverage concerns (25%, *n* = 66), scheduling issues (20%, *n* = 51), difficulty getting time off work (17%, *n* = 44), and transportation problems (16%, *n* = 41) were the most common barriers. Forty-six percent (*n* = 146) reported not having any barriers and 10% (*n* = 32) did not answer the question. For those who selected"other", two participants asked, “What is survivorship care?” and 16 participants commented on the distance and travel time to the facility (see Fig. 2).

**Table 2:** Survivorship clinic recommendations

	n	%
Number of topics to cover per visit		
One long visit to cover all topics	38	12.0%
Multiple shorter visits with less topics	142	45.0%
Other	110	35.0%
Missing	27	8.5%
Visit frequency		
Every 3 months	33	10.0%
Every 6 months	72	23.0%
Once per year	99	31.0%
Other	19	6.0%
Not Interested	73	23.0%
Missing	21	6.6%
Visit type*		
In person	190	60.0%
Telephone	123	39.0%
Video	120	38.0%
Support Groups	62	20.0%
None	57	18.0%
Missing	23	7.3%
*Multiple responses allowed

### Gender

We assessed differences by gender. In physical effects, females reported more dry mouth (51% vs 36%, *p* = 0.03), more unhappiness with face and neck appearance (27% vs 13%, *p* = 0.006), and trended toward more lymphedema that did not meet statistical significance (20% vs 12%, *p* = 0.089). Females had less sexual health concerns (2.5% vs 10%, *p* = 0.033). Other physical effects had no significant difference between genders (*p* > 0.14). There were no differences in other survivorship domains by gender (*p* > 0.13). When asked about interest in survivorship visits, more females vs. males expressed interest (56% vs 31%, *p* < 0.001). More females stated survivorship was a priority (51% vs 30%, *p* = 0.001), but both genders had low access to information on survivorship care (27% vs 32%, *p* = 0.4). In assessing preferred frequency of survivorship visits, females tended to desire more frequent visits (every three months 19% females vs 8.4% males; not interested in attending visits, 12% female vs 28% male, *p* = 0.011).

### Duration from treatment completion

We assessed responses based on the duration since treatment. Participants who more recently completed treatment had more lymphedema (24% vs 11%, *p* = 0.002) and had a trend toward more taste changes (31% vs 21%, *p* = 0.073), but fewer dental problems (13% vs 23%, *p* = 0.041). Regarding health promotion, patients who more recently completed treatment were less likely to feel that their providers discussed the importance of smoking cessation, diet, and exercise (28% vs 51%, *p* < 0.001). For chronic conditions, those that had more recently completed treatment felt less confident managing their chronic conditions with their primary care physician (11.6%) as compared to those further out from treatment completion (27%, *p* = 0.014). There were no significant differences in the psychosocial domain or the cancer recurrence domain.

When asked about survivorship visits, patients were equally likely to express interest in survivorship care regardless of length since treatment (45% ≤ 2 years since completion, 35% > 2 years since completion, *p* = 0.2). There was a trend toward more prioritization of survivorship for those who completed treatment more recently (45% ≤ 2 years since completion, 32% > 2 years since completion, *p* = 0.075). There was more access to information related to survivorship for those who recently completed treatment (44% ≤ 2 years since completion, 24% > 2 years since completion, *p* = 0.002). In assessing preferred frequency of survivorship visits, those who had completed treatment more recently tended to prefer more frequent survivorship visits (every three months, 19% ≤ 2 years since completion, 8.1% > 2 years since completion; every year, 22% ≤ 2 years since completion, 38% > 2 years since completion, *p* = 0.045).

## Discussion

Our survey provides an overview of the survivorship experience and needs of patients who completed RT for HNSCC, guided by a comprehensive survivorship framework. The survey highlighted several key areas of concern from cancer survivors themselves and provides guidance on patient preferences for a survivorship clinic. We used the comprehensive Nehklyudov survivorship framework to guide post-treatment management for cancer survivors [24], which underscores the need for not only standard cancer surveillance and physical effect management, but also acknowledges the additional domains of psychosocial, health promotion, and chronic conditions.

### Guideline-based survivorship needs

Management of physical side effects post-RT is an important component of oncologic care. Dry mouth emerged as the most common physical side effect, affecting 40% of respondents, with trouble swallowing, changes in taste, and dental issues also being significant concerns. Previous studies have shown that xerostomia impacts about 80% of patients with HNSCC post-RT, dysphagia affects approximately 50%, and dental caries occur in 30–37% [34–37]. These side effects varied by both gender and time since treatment. Females reported higher rates of dry mouth and lymphedema and were generally more dissatisfied with appearance than males. In contrast, males experienced more difficulty swallowing and pain in the head and neck area. Additionally, patients less than 2 years post-treatment reported higher rates of taste changes and lymphedema compared to those who were further out from treatment. These findings highlight the ongoing need for comprehensive side effect management, even in the very late (> 2 years) setting after treatment. Proven interventions, such as speech and swallow therapy, play a crucial role in addressing difficulties with voice, speech, and swallowing in HNSCC survivors post-radiation therapy [38]. Continuing to monitor, assess, and address late-onset effects ensures that survivors receive the support necessary to maintain their highest possible quality of life [39].

HNSCC diagnosis and treatment can affect survivors’ functionality, quality of life (QOL), mental and emotional well-being [16–19]. The psychosocial domain emerged as a significant gap, with 15% of survey participants meeting criteria for current depression and 71% feeling that mental health needs were not addressed during the cancer journey. The American Society of Clinical Oncology (ASCO) acknowledges the importance of screening for depression, anxiety, and other psychological effects in HNSCC survivors. Depression is now estimated to affect 15 to 50% of these survivors, and their suicide rate is two to three times higher than that of the general population or patients with other types of cancer [40–42]. Unfortunately, this is especially concerning for patients with HNSCC who experience functional losses, such as pain in the head and neck or difficulties with speech and swallowing [43, 44]. Efforts seeking to mitigate psychosocial impacts of HNSCC treatment and sequelae have included investigating the use of selective serotonin reuptake inhibitors to mitigate depression [45], seeking to mitigate body image disturbance and its impact on mental health including through the development of novel patient-reported outcome measures [46, 47], and investigating how HNSCC treatment sequela impacts sexual health [48, 49]. Further research seeking to mitigate this psychosocial impact is needed [50].

Within health promotion, while participants endorse individual components contributing to good health, over half of the patients did not comprehensively discuss all three topics of smoking, diet, and exercise with providers. Despite the American Cancer Society’s recommendations, there is a low prevalence of healthy lifestyle discussions among cancer patients and their providers. In a 2019 cross-sectional survey that asked 1,460 cancer survivors about conversations regarding healthy lifestyle topics, it was noted that just 17% of cancer survivors discussed all topics of health promotion with their providers [51]. Similarly, there has been limited research on interventions to improve health promotion in survivors of HNSCC; a systematic review in 2022 showed that the only intervention studied in this setting was Tai Chi Qigong’s effect on health promotion [52–54]. Further work on optimizing health-promoting behaviors after cancer treatment is needed, as certain lifestyle factors, such as diet, exercise, and environmental toxins, like smoking, can contribute to secondary cancer development [55].

Overall knowledge about survivorship was low. Two-thirds of participants indicated they did not have access to information on survivorship. This indicates an area of significant need, suggesting that implementation of a survivorship program will first need to focus on educating patients on the role of survivorship care while undergoing treatment. Despite detailed guidelines addressing survivorship care planning within the context of cancer care delivery, there remain gaps in communication regarding survivorship follow up [56–58]. The 2010 National Health Interview Survey revealed that one third of cancer survivors reported receiving treatment summaries with follow up recommendations and one third of oncologists reported conversations recommending survivorship care with their patients [59], similar to the findings in this current survey.

### Implications for survivorship program development

Survey participants provided guidance on how to structure future survivorship efforts. We demonstrate that patients prefer one longer visit covering all survivorship domains, rather than shorter visits addressing multiple topics. Over half of patients selected attending a survivorship visit every 6 months or every year, suggesting that patients may be scheduled for survivorship-specific visits outside of normal oncologic surveillance approximately every 6–12 months. Given the differences we show between patients based on time since treatment completion and patient preferences on frequency of survivorship clinics, at the 2-year mark, a comprehensive discussion with the patient could occur and future survivorship-focused follow-ups can be tailored to patient preferences and needs. We additionally showed that patients preferred to be seen in person. We hypothesize that seeing patients in person may aid in relationship building and could allow for better communication between patient and practitioner. When analyzing gender differences, there were no significant variations found beyond the physical effects domains, indicating that a survivorship clinic can be designed equally for both men and women.

Barriers to attending survivorship visits should be discussed with patients while on treatment and at their first routine follow-up. It is vital to recognize and address patient’s social needs early to prevent lapses in follow-up care [60, 61]. In this study, insurance coverage was the number one barrier to attending survivorship visits, followed by scheduling appointments around other visits and time off work. Insurance coverage and reimbursement depend on Medicare, Medicaid, and third-party payer benefit plans, therefore, ASCO advises verifying with the patient’s insurer to confirm which services are covered [62]. Follow-up visits tailored for survivorship can be coordinated with other oncologic-focused visits to minimize time off of work with the use of a nurse navigator [63].

Though there are survivorship clinics and guidelines developed in the past, to our knowledge, this is the first study that provides patient insight on developing a comprehensive structure for survivorship care guided by a framework. Future work investigating efforts to optimize survivorship care for patients in the context of the comprehensive Nekhlyudov domains should also include the broader contextual domains of the framework [24], which were out of scope of this current study. These contextual domains include healthcare delivery structure, care coordination, and sociodemographic or community characteristics, all of which may impact the overall survivorship experience and access to care. This survey study also confirms a shortfall of communication and education surrounding survivorship care between patients and providers, potentially contributing to less access to survivorship care.

### Limitations

Limitations of this study include its cross-sectional design and the risk of recall bias, as participants were asked to reflect on treatment experiences that may have occurred years earlier. Additionally, the study population was predominantly older, white men, with voices of women and minorities being under-represented in the survey findings. The response rate of 28% may limit generalizability to broader and more diverse populations. We contacted patients through email addresses collected in the electronic medical record system. This method may have unintentionally omitted patients with less literacy or internet access, who may not have used email to communicate with their healthcare system. We asked patients about preferences for a hypothetical survivorship visit, which may not reflect true patient preferences once in a clinic setting. Ongoing work extending our findings to a more diverse group of patients and validating patient preferences with implementation-focused work is needed to optimize survivorship care. Finally, this single snapshot of survivorship needs in a group of patients who completed treatment over a period of time does not capture evolving survivorship over time, and additional work is needed to understand how to adapt to evolving survivorship needs.

## Conclusion

Our study highlights the multifaceted needs of HNSCC survivors, emphasizing the importance of a tailored, patient-centered approach to survivorship care. By addressing the five domains in the Cancer Survivorship Framework and barriers through CFIR guidance, healthcare providers can better support the long-term well-being of cancer survivors. These findings provide a foundation for developing robust survivorship care programs that meet the diverse needs of this patient population.

## Supplementary Information

Below is the link to the electronic supplementary material.

Supplementary file1 (DOCX 47 KB)

## Data Availability

Data are available upon reasonable request to the corresponding author.
