# Correction to: Survivorship therapy needs after radiotherapy for head and neck cancer: surveying opportunities for growth (STRONG)

**DOI:** 10.1007/s00520-025-09644-x

**Published:** 2025-06-12

**Authors:** Alexis Larson, Yangruijue Ma, Zequn Sun, Janine Kingsbury, Katelyn O. Stepan, Adil Akthar, Jochen Lorch, Bharat B. Mittal, Poonam Yadav, Michelle L. Mierzwa, Leila J. Mady, Laila A. Gharzai

**Affiliations:** 1 Department of Radiation Oncology, Northwestern University, 251 East Huron Street, Ste LC‑ 178, Chicago, IL 60611 USA; 2 Department of Biostatistics, Northwestern University, Chicago, IL USA; 3 Department of Otolaryngology, Northwestern University, Chicago, IL USA; 4 Division of Medical Oncology, Department of Medicine, Northwestern University, Chicago, IL USA; 5 Department of Radiation Oncology, University of Michigan, Ann Arbor, MI USA; 6 Department of Otolaryngology, Johns Hopkins University, Baltimore, MD USA; 7 Department of Medical Social Sciences, Northwestern University, Chicago, IL USA

**Correction to: Supportive Care in Cancer (2025) 33:403.**

10.1007/s00520-025-09429-2

The correct name of the second author is Yangruijue Ma, not Yangruijue Anna.

The original article has been corrected.

