# Supplementary material for: Survivorship therapy needs after radiotherapy for head and neck cancer: surveying opportunities for growth (STRONG)
Source: Support Care Cancer. 2025 Apr 29;33(5):403. doi: 10.1007/s00520-025-09429-2 (PMC12041175; doi:10.1007/s00520-025-09429-2)
Supplement: Supplementary file 1 — Supplementary file1 (DOCX 47 KB) [file 520_2025_9429_MOESM1_ESM.docx]

**Supplementary Materials**

Larson et al. Survivorship Therapy needs after RadiOtherapy for head and Neck cancer: surveying opportunities for Growth (STRONG). *Supportive Care in Cancer.* 2025.

[TABLES 1](#_Toc191473073)

[Supplementary Table 1. Multivariable analysis of three domains from the Consolidated Framework for Implementation Research (CFIR): individual domain (assessing interest in survivorship), inner setting process domain (access to information in survivorship), implementation process domain (considering survivorship as a priority). 1](#_Toc191473074)

[FIGURES 2](#_Toc191473075)

[Supplementary Figure 1. Five cancer survivorship domains and overlying contexts affecting implementation, adapted from Nekhlyudov et al (JNCI 2019). 2](#_Toc191473076)

[Supplementary Figure 2. Flow diagram of study patients. 3](#_Toc191473077)

# TABLES

## Supplementary Table 1. Multivariable analysis of three domains from the Consolidated Framework for Implementation Research (CFIR): individual domain (assessing interest in survivorship), inner setting process domain (access to information in survivorship), implementation process domain (considering survivorship as a priority).

|  | Interest in Survivorship Care | | Survivorship Care is a Priority | | Access to Survivorship Care | |
| --- | --- | --- | --- | --- | --- | --- |
| Variable | OR (95% CI) | p-value | OR (95% CI) | p-value | OR (95% CI) | p-value |
| Age (per 10 years) | 0.91 (0.72, 1.16) | 0.5 | 1.06 (0.83, 1.35) | 0.7 | 1.07 (0.83, 1.39) | 0.6 |
| Gender (referent = male) |  |  |  |  |  |  |
| Female | 2.67 (1.48, 4.86) | **0.001** | 2.79 (1.54, 5.11) | **<0.001** | 0.91 (0.47, 1.72) | 0.8 |
| Race (referent = White) |  |  |  |  |  |  |
| Non-White | 1.65 (0.65, 4.22) | 0.3 | 1.80 (0.7, 4.66) | 0.2 | 1.17 (0.41, 3.13) | 0.8 |
| Surgery received | 1.05 (0.54, 2.07) | 0.9 | 1.06 (0.55, 2.09) | 0.9 | 2.50 (1.24, 5.29) | **0.013** |
| Chemotherapy received | 0.78 (0.44, 1.37) | 0.4 | 0.82 (0.46, 1.46) | 0.5 | 2.09 (1.15, 3.85) | **0.016** |
| Duration since Radiation (referent = ≤ 2 years) |  |  |  |  |  |  |
| > 2 years | 0.58 (0.32, 1.04) | 0.068 | 0.52 (0.29, 0.93) | **0.027** | 0.34 (0.19, 0.62) | **<0.001** |
| Currently undergoing cancer treatment | 2.55 (0.92, 7.26) | 0.072 | 2.31 (0.84, 6.57) | 0.11 | 3.01 (1.08, 8.83) | **0.038** |
| Cancer recurrence | 1.09 (0.47, 2.48) | 0.8 | 1.31 (0.56, 2.98) | 0.5 | 0.81 (0.33, 1.89) | 0.6 |

# FIGURES

## Supplementary Figure 1. Five cancer survivorship domains and overlying contexts affecting implementation, adapted from Nekhlyudov et al (JNCI 2019).

INDIVIDUAL DOMAIN

INTERPERSONAL FACTORS

COMMUNITY FACTORS

## Supplementary Figure 2. Flow diagram of study patients.
